# Development of a nomogram for predicting 90-day mortality in patients with sepsis-associated liver injury

**DOI:** 10.1038/s41598-023-30235-5

**Published:** 2023-03-04

**Authors:** Liangwen Cui, Junjie Bao, Chao Yu, Cheng Zhang, Rui Huang, Lian Liu, Min Shao

**Affiliations:** 1 Department of Critical Care Medicine, The First Affiliated Hospital of Anhui Medical University, Jixi Road 218, Hefei, 230022 Anhui China; 2 Department of Anhui Provincial Cancer Institute, The First Affiliated Hospital of Anhui Medical University, Jixi Road 218, Hefei, 230022 China

**Keywords:** Diseases, Medical research, Risk factors

## Abstract

The high mortality rate in sepsis patients is related to sepsis-associated liver injury (SALI). We sought to develop an accurate forecasting nomogram to estimate individual 90-day mortality in SALI patients. Data from 34,329 patients were extracted from the public Medical Information Mart for Intensive Care (MIMIC-IV) database. SALI was defined by total bilirubin (TBIL) > 2 mg/dL and the occurrence of an international normalized ratio (INR) > 1.5 in the presence of sepsis. Logistic regression analysis was performed to establish a prediction model called the nomogram based on the training set (n = 727), which was subsequently subjected to internal validation. Multivariate logistic regression analysis showed that SALI was an independent risk factor for mortality in patients with sepsis. The Kaplan‒Meier curves for 90-day survival were different between the SALI and non-SALI groups after propensity score matching (PSM) (log rank: P < 0.001 versus P = 0.038), regardless of PSM balance. The nomogram demonstrated better discrimination than the sequential organ failure assessment (SOFA) score, logistic organ dysfunction system (LODS) score, simplified acute physiology II (SAPS II) score, and Albumin–Bilirubin (ALBI) score in the training and validation sets, with areas under the receiver operating characteristic curve (AUROC) of 0.778 (95% CI 0.730–0.799, P < 0.001) and 0.804 (95% CI 0.713–0.820, P < 0.001), respectively. The calibration plot showed that the nomogram was sufficiently successful to predict the probability of 90-day mortality in both groups. The DCA of the nomogram demonstrated a higher net benefit regarding clinical usefulness than SOFA, LODS, SAPSII, and ALBI scores in the two groups. The nomogram performs exceptionally well in predicting the 90-day mortality rate in SALI patients, which can be used to assess the prognosis of patients with SALI and may assist in guiding clinical practice to enhance patient outcomes.

## Introduction

Sepsis is one of the common causes of death in patients who are admitted to the ICU^1,2^. Many clinical investigations have mentioned that liver failure in patients with sepsis is rapidly progressive and has high morbidity and mortality rates^3–6^. The pathophysiological mechanism of sepsis-associated liver injury (SALI) has not been clearly elucidated, and it may be relevant to the inflammatory cellular response, direct damage by endotoxin, hepatic microcirculatory dysfunction and disturbance of bilirubin-bile acid metabolism^7^. Sepsis-induced ischemic-hypoxic liver injury and sepsis-associated cholestasis are common manifestations of SALI. Although there is no unified SALI standard, the current recommended standard is largely based on total bilirubin (TBIL) > 2 mg/dL and the development of international normalized ratio (INR) > 1.5^8,9^. The albumin-bilirubin (ALBI) score is a novel liver function evaluation score that has excellent liver function assessment and prognosis prediction performance and has also been widely used to assess other liver diseases in recent years^10–12^.

The ALBI score had better performance than the Child‒Pugh scale and the MELD score in forecasting morbidity and mortality rates in patients with liver disease^13,14^. Meanwhile, the ALBI score has not been used to assess liver injury in sepsis to date. The SOFA score, LODS score and SAPS II score often have a considerable ability to predict sepsis in patients but rarely in SALI patients^15^. The high mortality of SALI patients may be related to the lack of available diagnostic tools, especially those capable of accounting for the predictive factors of sepsis and early-onset liver injury^15^. The nomogram has been widely used as a visualization tool for clinical prognostic studies in survival studies of critically ill patients^16–18^. Accordingly, it is necessary to positively explore a nomogram that can effectively predict liver injury in sepsis.

## Materials and methods

### Data origin

The Medical Information Mart for Intensive Care IV (MIMIC-IV) database is a public database that incorporates detailed patient information from patients hospitalized at Beth Israel Deaconess Hospital (Bowers, Massachusetts, USA) between 2008 and 2019. A retrospective study was performed using the MIMIC-IV (v 2.21) database. The database is constantly updated with the latest version (v2.21) released on 12 July 2022, and more data have been added to increase the comprehensiveness of the data. Patient consent is not required because the identifying information in this platform is hidden. The investigators are enrolled in a learning program offered by the National Institutes of Health (NIH) and are granted the freedom to access the MIMIC-IV database after passing the requisite assessments (certificate number 40655812). All methods were carried out in accordance with relevant guidelines and regulations. All experimental protocols were approved by the Institutional Review Boards of both Beth Israel Deaconess Medical Center (Boston, MA, USA) and the Massachusetts Institute of Technology (Cambridge, MA, USA). Since all data de-identified in this database to remove patients’ information, the requirement for individual patient consent is not indispensable.

### Research population and data extraction

Navicat Premium (version 15.0.12, Hong Kong, China) was developed as an intermediate software to connect with the MIMIC IV database, and the requested information was obtained by structure query language (SQL). We included patients who were above the age of 17 and had been diagnosed with sepsis according to internationally recognized Sepsis-3 criteria (ICD-9 code 99591 and ICD-9 code 99592): (1) Patients with pathogenically positive infections and Sequential Organ Failure Assessment (SOFA) scores ≥ 2^1^.

**Figure 1:**
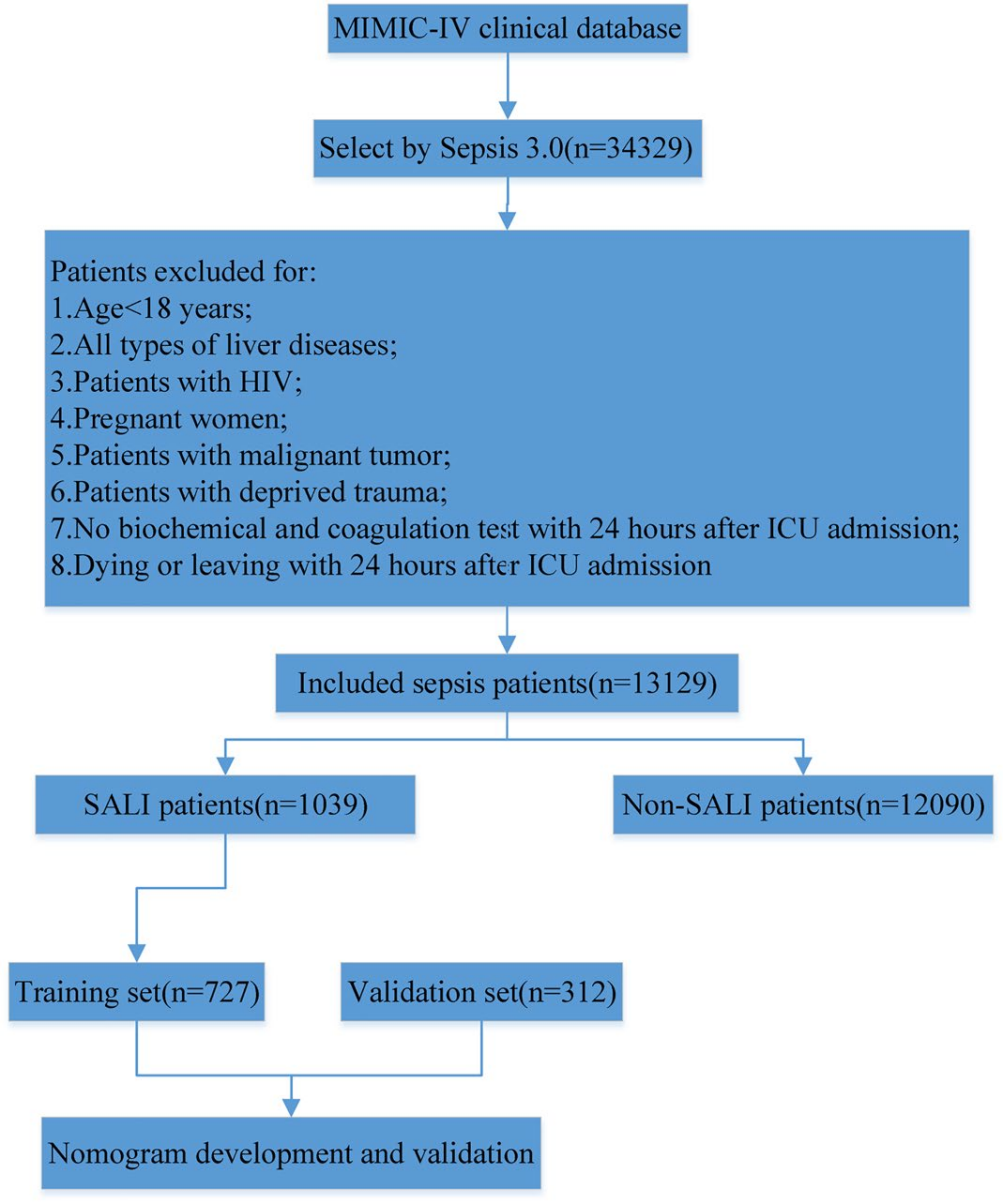
Flowchart of data extraction and study design.

The study extracted relevant variables from the MIMIC-IV database: (1) basic information about the patient, including age and gender; (2) comorbidity; (3) severity score, including Sequential Organ Failure Assessment (SOFA), Logistic Organ Dysfunction System (LODS), and Simplified Acute Physiology II (SAPSII) score; (4) mean vital signs and worst laboratory results within 24 h in the ICU; (5) infection site; and (6) outcomes, including ICU stay time, hospital stay time, the ratio of 90-day mortality, and the first day of ICU usage. Patients were excluded for: (1) age < 18 years; (2) all types of liver diseases; (3) patients with HIV; (4) pregnant women; (5) patients with malignant tumor; (6) patients with deprived trauma; (7) no biochemical and coagulation test with 24 h after ICU admission; (8) death or discharge from hospital with 24 h after ICU admission. A total of 13,129 eligible patients were ultimately included in our study cohort (Fig. 1).

### Sepsis-associated liver injury

Based on all eligible patients with sepsis, SALI patients were defined as admission to the ICU for the worst 24-h value total bilirubin (TBIL) > 2 mg/dL and the development of an international normalized ratio (INR) > 1.5^8,9^. Logistic regression was performed on the parameters of eligible SALI patients to generate the predictive model. Data extraction and research concepts are shown in Fig. 1.

### Statistical analysis

Continuous variables were represented using the median (IQR) and were measured by the Mann‒Whitney U test. Categorical information was described in terms of numbers (percentages) and was tested by the χ^2^ test. Parameters that were valid for univariate analysis were taken into account in the multivariate logistic analysis, and their odds ratios (ORs) were calculated. When the VIF was ≤ 2, then a noncollinear relationship between the two groups was present. The 90-day survival rates between the SALI and non-SALI groups were assessed by Kaplan‒Meier analysis, and the log rank test was performed to determine the difference between the two groups, with P < 0.05 considered statistically significant. To address the baseline imbalance, we conducted a sample analysis using propensity score matching (PSM) and calculated the differences in 90-day survival times between SALI patients and non-SALI patients.

SALI patients were randomized into training and validation cohorts at a ratio of 7:3. Statistically significant clinical variables from the training cohort were included in a multivariate logistic regression analysis with reverse stepwise selection using the likelihood ratio test and Akaike information criterion (AIC) as a stopping rule^19^. The multivariate analysis regression results from the training cohort were used to develop a nomogram to assess 90-day mortality according to Occam’s Razor, which means that a successful model should have a minimum number of metrics to illustrate the most research targets in the study^20^. The calibration of 1000 bootstrap resamples was assessed for the predictability of the nomogram, and Harrell’s concordance index (C-index) was used to determine the predictive performance of the model.

The study compared the performance of the SOFA, LODS, SAPS II and ALBI scores with the expression of the nomogram using an area under the curve of the receiver operating characteristic (AUROC) and decision curve analysis (DCA). In addition, integrated discrimination improvement (IDI) and net reclassification improvement (NRI) indices were applied to enhance the predictive model and the accuracy of the predictive model.

Stata software (Stata/MP V.17, Texas, USA) and R software (V 4.2.1, Vienna, Austria) were used for statistical analysis. All parameters with ≥ 30% missing data were excluded. For missing data, we used the mice package to obtain 5 packets by multiple interpolation and merged them. In addition, we also used packages such as MatchIt, pROC, survival, survminer, Formula, ggplot2, Hmisc, glmnet, forestplot and IswR in R software. All analyses were performed according to the Transparent Reporting of Multivariate Predictive Models for Individual Prognosis or Diagnosis (TRIPOD) guidelines, which refer to the principles and rationale for analysis^21^.

### Ethics statements

There is not any human participants who are involve in the study. Our experiment was performed in accordance with relevant guidelines and regulations.

### Ethics approval and consent to participate

Patient consent is not required because the identifying information in this platform is hidden. The investigators are enrolled in a learning program offered by the National Institutes of Health (NIH) and are granted the freedom to access the MIMIC-IV database after passing the requisite assessments (certificate number 40655812).

## Results

### Clinical characteristics

We enrolled a total of 34,329 patients with sepsis from the MIMIC-IV clinical database, and subsequently screened 13,129 patients with sepsis based on inclusion and exclusion criteria. In this study, 1039 SALI patients were finally enrolled. Age, baseline information, mean value of test results, worst value of vital signs and results during the first day in the ICU between SALI patients and non-SALI patients are shown in Table 1. SALI patients have a median age of 65 (51, 77) years, and non-SALI patients have a higher median age of 68 (56, 79) years. Regarding comorbidities, we observed that SALI patients had lower rates of myocardial infarction, chronic pulmonary disease, cerebrovascular disease, paraplegia, and renal disease than non-SALI patients but were more likely to have congestive heart failure, peripheral vascular disease, rheumatic disease, and peptic ulcer than non-SALI patients. Additionally, the overall severity level of diseases was worse in SALI patients than in non-SALI patients, and the differences were statistically significant (P < 0.001), including the SOFA score, LODS score and SAPS II score.

**Table 1:** The characteristics of patients included at first ICU admission.

Variable	All patients (n = 13,129)	Non-SALI patients (n = 12,090)	SALI patients (n = 1039)	P value
Age, years	68 (56, 79)	68 (56, 79)	65 (51, 77)	< 0.001
Gender, male	7369 (56.13)	6741 (55.76)	628 (60.44)	0.004
Comorbidity, n (%)				
Myocardial infarct	2772 (21.81)	2683 (22.19)	181 (17.42)	< 0.001
Congestive heart failure	5261 (40.07)	4819 (39.86)	442 (42.54)	0.097
Chronic pulmonary disease	3753 (28.59)	3502 (28.97)	251 (24.16)	0.001
Peripheral vascular disease	1868 (14.23)	1718 (14.21)	150 (14.44)	0.877
Cerebrovascular disease	2119 (16.14)	2033 (16.82)	86 (8.28)	< 0.001
Diabetes	1804 (13.74)	1712 (14.16)	92 (8.85)	< 0.001
Rheumatic disease	555 (4.23)	519 (4.29)	36 (3.46)	0.233
Peptic ulcer	481 (3.66)	440 (3.64)	41 (3.95)	0.675
Paraplegia	762 (5.86)	733 (6.06)	29 (2.79)	< 0.001
Renal disease	3882 (29.57)	3606 (29.83)	276 (26.56)	0.030
Severity score				
SOFA score	3.00 (2.00, 5.00)	3.00 (2.00, 4.00)	5.00 (3.00, 7.00)	< 0.001
LODS score	6.00 (4.00, 9.00)	6.00 (4.00, 9.00)	8.00 (5.00, 11.00)	< 0.001
SAPS II score	39.00 (31.00, 49.00)	39.00 (31.00, 45.00)	46.00 (35.00, 58.00)	< 0.001
First day of ICU usage^a^				
First day CRRT, n (%)	730 (5.56)	633 (5.24)	97 (9.34)	< 0.001
First day vasopressor, n (%)	6447 (49.11)	5770 (44.73)	677 (65.16)	< 0.001
First day MV, n (%)	5632 (42.90)	5120 (42.35)	512 (49.28)	< 0.001
Vital signs^b^				
Mean heart rate (min^−1^)	103.00 (89.00, 119.00)	102.00 (88.00, 118.00)	108.00 (93.00, 124.00)	< 0.001
Mean arterial pressure (mmHg)	59.00 (52.00, 67.00)	59.00 (52.00, 67.00)	57.00 (49.00, 65.00)	< 0.001
Mean respiratory rate (min^−1^)	27.00 (23.00, 32.00)	27.00 (23.00, 32.00)	28.00 (24.00, 33.00)	< 0.001
Mean temperature (°C)	37.20 (36.83, 37.78)	37.22 (36.83, 37.80)	37.06 (36.72, 37.67)	< 0.001
Mean spo2 (%)	93.00 (90.00, 96.00)	93.00 (91.00, 96.00)	93.00 (90.00, 95.00)	< 0.001
Blood gas analysis^c^				
PH-min	7.32 (7.23, 7.39)	7.32 (7.23, 7.39)	7.27 (7.16, 7.36)	< 0.001
PO2-min, (mmHg)	86.00 (68.00, 115.00)	86.00 (68.00, 116.00)	82.00 (66.00, 106.00)	< 0.001
PCO2-max, (mmHg)	45.00 (38.00, 53.00)	45.00 (38.00, 54.00)	43.00 (36.00, 52.00)	< 0.001
Lactate_max, (mmol/L)	2.20 (1.40, 4.20)	2.10 (1.30, 3.80)	4.70 (2.60, 8.30)	< 0.001
Bicarbonate_min, (mEq/L)	21.00 (17.00, 24.00)	21.00 (18.00, 24.00)	18.00 (15.00, 22.00)	< 0.001
Laboratory tests^c^				
Glucose_max, (mg/dL)	157.00 (123.00, 218.00)	158.00 (124.00, 218.00)	153.00 (118.00, 216.00)	0.033
Aniongap_max	17.00 (14.00, 21.00)	17.00 (14.00, 20.00)	19.00 (16.00, 24.00)	< 0.001
Chloride_max, (mEq/L)	106.00 (102.00, 110.00)	106.00 (102.00, 110.00)	105.00 (100.00, 110.00)	< 0.001
Potassium_max, (K/uL)	4.50 (4.10, 5.10)	4.50 (4.10, 5.10)	4.60 (4.10, 5.20)	0.178
Sodium_min, (mEq/L)	140.00 (137.00, 143.00)	140.00 (137.00, 143.00)	140.00 (136.00, 143.00)	< 0.001
Calcium_min, (mg/dL)	7.90 (7.40, 8.50)	7.90 (7.40, 8.50)	7.70 (7.10, 8.20)	< 0.001
Indicators for kidney function^c^				
BUN_max, (mg/dL)	28.00 (18.00, 47.00)	28.00 (18.00, 47.00)	34.00 (20.00, 55.00)	< 0.001
Creatinine _max, (mg/dL)	1.40 (0.90, 2.30)	1.30 (0.90, 2.30)	1.70 (1.10, 2.70)	< 0.001
Indicators for liver function^c^				
TBIL_max (mg/dL)	0.70 (0.40, 1.20)	0.60 (0.40, 1.00)	3.60 (2.60, 5.90)	< 0.001
ALT_max (U/L)	28.00 (16.00, 67.00)	27.00 (16.00, 57.00)	105.50 (36.00, 432.00)	< 0.001
AST_max (U/L)	42.00 (25.00, 101.00)	39.00 (24.00, 85.00)	163.00 (69.00, 644.00)	< 0.001
ALP_max (U/L)	87.00 (63.00, 131.00)	85.00 (62.00, 125.00)	132.00 (80.25, 228.75)	< 0.001
Albumin_min (g/dL)	3.10 (2.60, 3.60)	3.10 (2.70,3.60)	2.80 (2.30, 3.20)	< 0.001
Blood routine examination^c^				
WBC_max, (K/uL)	14.20 (10.10, 19.70)	14.10 (10.10, 19.50)	15.80 (10.40, 22.40)	< 0.001
Hemoglobin_min (g/dL)	9.60 (8.10, 11.30)	9.70 (8.20, 11.30)	9.10 (7.70, 10.90)	< 0.001
Platelet _min (K/uL)	171.00 (117.00, 238.00)	175.00 (123.00, 243.00)	111.00 (66.00, 168.00)	< 0.001
RDW_max, (%)	15.20 (14.00, 16.70)	15.10 (13.90, 16.60)	16.60 (14.90, 18.45)	< 0.001
MCV_min, (fL)	91.00 (87.00, 95.00)	91.00 (87.00, 95.00)	91.00 (86.00, 96.00)	0.715
MCH_min, (pg)	30.00 (28.40, 31.40)	30.00 (28.40, 31.30)	30.30 (28.70, 31.80)	< 0.001
MCHC_min, (g/L)	32.70 (31.60, 33.80)	32.70 (31.60, 33.80)	33.10 (32.00, 34.20)	< 0.001
Indicators for coagulation function^c^				
INR_max	1.40 (1.20, 1.80)	1.30 (1.20, 1.60)	2.20 (1.80, 3.20)	< 0.001
APTT_max, (s)	34.20 (28.90, 48.70)	33.50 (28.60, 46.70)	44.50 (35.70, 65.10)	< 0.001
Infection site, n (%)				
Lung	2373 (18.65)	2279 (19.46)	94 (9.23)	< 0.001
Abdominal	170 (1.34)	159 (1.36)	11 (1.08)	0.550
Urinary tract	343 (2.70)	336 (2.87)	7 (0.69)	< 0.001
SSTI	14 (0.11)	13 (0.11)	1 (0.00)	1.000
Bacteremia	9771 (76.77)	8867 (75.73)	904 (88.80)	< 0.001
Outcome				
ICU stay time, days	3.75 (2.08, 7.58)	3.75 (2.08, 7.46)	4.17 (2.21, 8.88)	0.002
Hospital stay time, days	10.00 (6.00, 17.00)	10.00 (6.00, 17.00)	11.00 (6.00, 20.00)	< 0.001
90-day mortality, n (%)	3827 (29.15)	3369 (27.87)	458 (44.08)	< 0.001
CRRT, n (%)	1124 (8.56)	906 (7.49)	218 (20.98)	< 0.001
Vasopressor, n (%)	7189 (54.76)	6459 (53.42)	730 (70.26)	< 0.001
MV, n (%)	7260 (55.30)	6649 (55.00)	611 (58.81)	0.019

Categorical data are presented as frequencies (percentages), and parametric continuous data are presented as medians (interquartile ranges).

*SOFA score* sequential organ failure assessment score, *LODS score* logistic organ dysfunction system score; *SAPS II score* simplified acute physiology II score, *PH* potential of hydrogen, *PO2* arterial partial pressure of oxygen, *PCO2* arterial blood carbon dioxide partial pressure, *BUN* blood urea nitrogen, Creatinine serum creatinine, *TBIL* total bilirubin, *ALT* alanine aminotransferase, *AST* aspartate aminotransferase, *ALP* alkaline phosphatase, *WBC* white blood cell, *RDW* red blood cell distribution width, *MCV* mean corpuscular volume, *MCH* mean corpuscular hemoglobin, *MCHC* mean corpuscular hemoglobin concentration, *INR* international normalized ratio, *APTT* partial thromboplastin time, *SSTI* skin and soft tissue infection.

^a^First day of ICU usage means the number of ICU uses on the first day; *CRRT* continuous renal replacement therapy, *MV* mechanical ventilation.

^b^Vital signs were calculated as the mean value during each patient’s first 24 h after ICU admission.

^c^The laboratory tests recorded the worst value during each patient's first 24 h from ICU admission.

We also observed a considerable increase in the use of continuous renal replacement therapy (CRRT), vasopressors and mechanical ventilation (MV) during the first 24 h in the ICU in SALI patients, all P < 0.001. In addition, the majority of SALI patients had worse vital signs and laboratory parameters. The proportion of lung and urinary tract infections was high in patients with non-SALI, but the proportion of bacteremia infections was higher in SALI patients than in non-SALI patients. Last, in the study endpoints, outcomes, such as ICU stay time and hospital stay time, were longer in SALI patients, and the number of deaths in 90 days for patients who used vasopressors, CRRT and MV was higher.

### SALI is an independent risk factor for 90-day mortality in septic patients

After the correction, SALI was detected as an independent risk indicator for 90-day mortality in patients with sepsis by logistic multivariate analysis [odds ratio (OR) 1.158, 95% CI 0.970–1.240, P = 0.034] (Supplementary Table 1). Our data were matched using propensity score matching (PSM) because the SALI patient group and the non-SALI patient group had significant discrepancies. After matching, the number of patients in the matched and unmatched groups was 1039. The 90-day Kaplan–Meier survival analysis was performed before and after matching, with log-rank P < 0.001 before PSM and P = 0.038 after PSM. Consequently, there was a substantial distinction in 90-day mortality between the SALI patients and non-SALI patients, whether matching was performed or not (Supplementary Fig. 1).

### Construction of the nomogram in the training set

**Figure 2:**
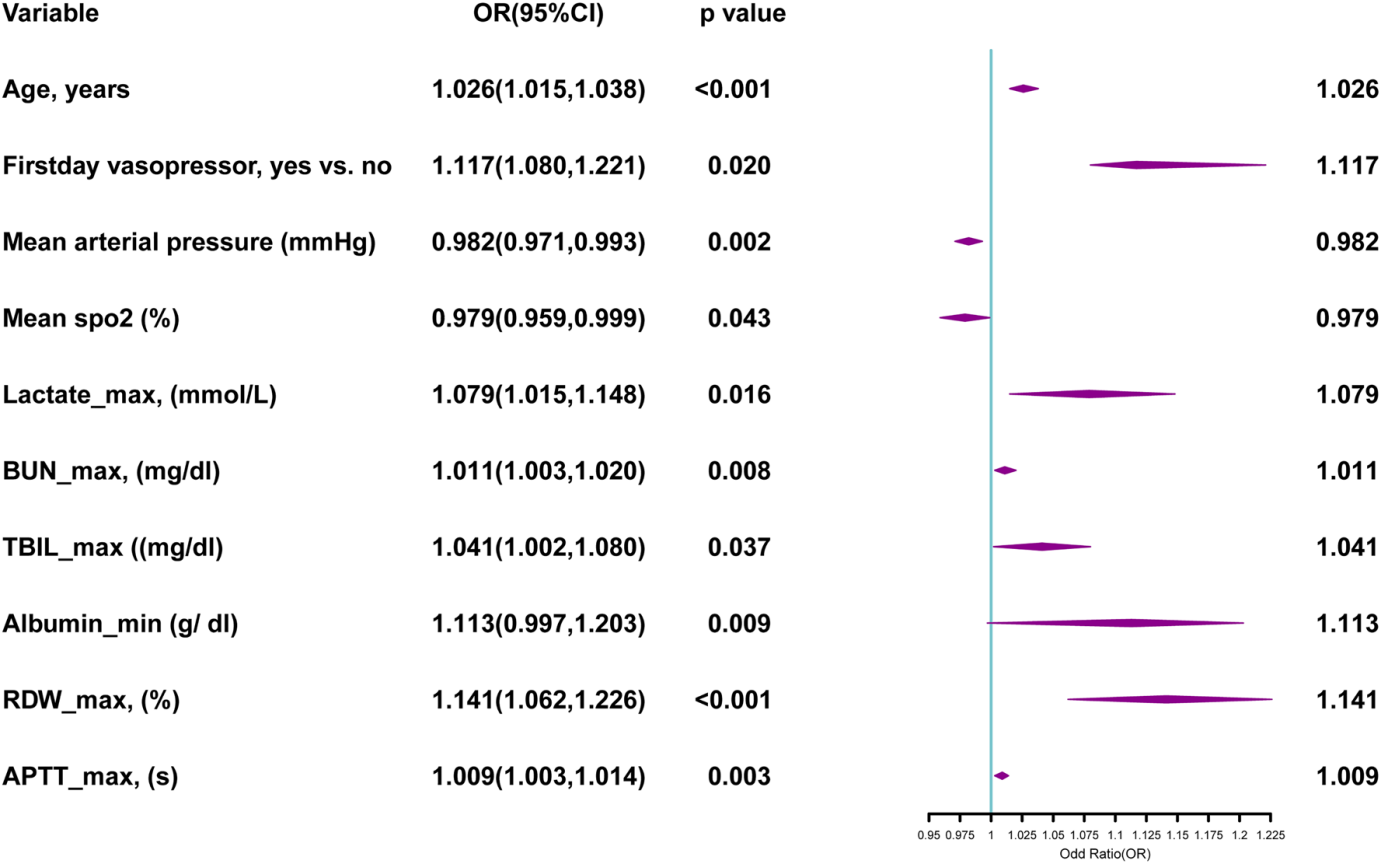
Factors independently associated with 90-day mortality in SALI patients in the training cohort by multivariate logistic regression analysis with forest plots.

**Figure 3:**
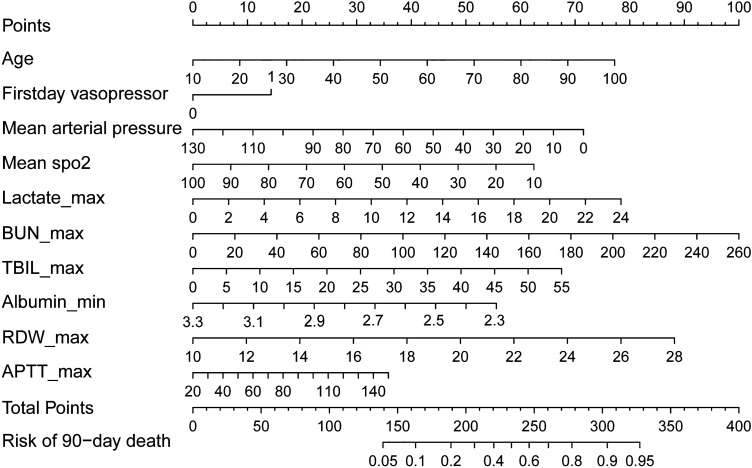
Nomogram for estimating the risk of 90-day mortality in SALI patients. By developing the scale score, the change in each variable is described by the forest plot, and then the total score is computed to predict the possibility that an event will happen.

After a series of screenings, resulting in 1039 SALI patients, a random allocation of 70% was given as the training set and the other 30% as the validation set, 727 and 312, respectively. There was no noticeable difference between the training and validation sets with the exception of the skin and soft tissue infection (SSTI) parameter (Supplementary Table 2). Our research was performed based on a training set of 727 patients. In univariate analysis, basic patient information, comorbidities, first day of ICU use, mean vital signs during 24 h in the ICU, worst laboratory results and site of infection were screened for statistically significant parameters that were included in the logistic multivariate analysis. And parameters finally were added to the multivariate logistic regression analysis, including age, the use of vasopressors, mean arterial pressure, mean oxygen saturation (spo2), lactate maximum, blood urea nitrogen (BUN) maximum, total bilirubin (TBIL) maximum, albumin minimum, red blood cell distribution width (RDW) maximum and activated partial thromboplastin time (APTT) maximum, on which the forest plots were charted (Table 2, Fig. 2). A total of 10 variables illustrated in the forest plot above all followed the AIC principle, and all of the continuous variables had a VIF ≤ 2, so that there were covariance variables in Fig. 2. The nomogram is based on multivariate logistic regression analysis in which the 10 indicators, and are then plotted on the same plane with scaled lines to represent the interrelationships between the variables in the prediction model. The predictive value of the outcome event is calculated by converting the total score into a function of the probability of the outcome event. Nomogram was generated to determine the propensity of 90-day mortality in SALI patients (Fig. 3).

**Table 2:** Factors independently associated with 90-day mortality of patients with SALI by univariate logistic regression analysis in the training cohort.

Variable	OR (95% CI)	P value
Age, years	1.026 (1.017, 1.035)	< 0.001
Gender, male	1.029 (0.761, 1.391)	0.852
Myocardial infarct	1.953 (1.329, 2.870)	0.001
Congestive heart failure	1.640 (1.218, 2.209)	0.001
Chronic pulmonary disease	1.263 (0.903, 1.767)	0.173
Peripheral vascular disease	1.683 (1.100, 2.575)	0.016
Cerebrovascular disease	1.028 (0.618, 1.708)	0.916
Diabetes	1.949 (1.187, 3.200)	0.008
Rheumatic disease	0.965 (0.432, 2.156)	0.931
Peptic ulcer	1.339 (0.620, 2.891)	0.457
Paraplegia	0.609 (0.206, 1.800)	0.370
Renal disease	1.642 (1.179, 2.287)	0.003
First day CRRT, yes vs. no	2.364 (1.387, 4.030)	0.002
First day vasopressor, yes vs. no	1.834 (1.335, 2.518)	< 0.001
First day MV, yes vs. no	1.106 (0.995, 1.129)	< 0.001
Mean heart rate (min^−1^)	1.004 (0.998, 1.010)	0.239
Mean arterial pressure (mmHg)	0.973 (0.964, 0.983)	< 0.001
Mean respiratory rate (min^−1^)	1.013 (0.994, 1.034)	0.188
Mean temperature (°C)	0.718 (0.602, 0.856)	< 0.001
Mean spo2 (%)	0.967 (0.949, 0.985)	< 0.001
PH-min	0.275 (0.095, 0.799)	0.018
PO2-min, (mmHg)	0.995 (0.991, 0.999)	0.016
PCO2-max, (mmHg)	0.995 (0.985, 1.005)	0.320
Lactate_max, (mmol/L)	1.110 (1.068, 1.153)	< 0.001
Bicarbonate_min, (mEq/L)	0.943 (0.918, 0.968)	< 0.001
Laboratory tests		
Glucose_max, (mg/dL)	1.001 (0.999, 1.002)	0.276
Aniongap_max, (mg/dL)	1.073 (1.048, 1.100)	< 0.001
Chloride_max, (mEq/L)	0.986 (0.967, 1.005)	0.138
Potassium_max, (K/uL)	1.364 (1.167, 1.595)	< 0.001
Sodium_min, (mEq/L)	1.010 (0.987, 1.034)	0.392
Calcium_min, (mg/dL)	1.041 (0.894, 1.212)	0.605
BUN_max, (mg/dL)	1.019 (1.013, 1.025)	< 0.001
Creatinine _max (mg/dL)	1.238 (1.117, 1.371)	< 0.001
TBIL_max (mg/dL)	1.032 (1.002, 1.062)	0.036
ALT_max (U/L)	1.000 (1.000, 1000)	0.480
AST_max (U/L)	1.000 (1.000, 1000)	0.222
ALP_max (U/L)	1.001 (1.000, 1.002)	0.035
Albumin_min (g/dl)	1.082 (0.970, 1.180)	0.008
WBC_max, (K/uL)	0.994 (0.982, 1.007)	0.358
Hemoglobin_min (g/dL)	0.939 (0.880, 1.002)	0.056
Platelet_min (K/uL)	0.998 (0.996, 1.000)	0.035
RDW_max, (%)	1.177 (1.110, 1.248)	< 0.001
MCV_min, (fL)	1.010 (0.993, 1.027)	0.254
MCH_min, (pg)	0.968 (0.922, 1.018)	0.204
MCHC_min, (g/L)	0.899 (0.825, 0.981)	0.016
INR_max	1.119 (1.034, 1.211)	0.005
APTT_max, (s)	1.010 (1.006, 1.015)	< 0.001
Infection site, n (%)		
Lung	0.857 (0.508, 1.447)	0.564
Abdominal	1.323 (0.352, 4.969)	0.678
Urinary tract	0.525 (0.095, 2.883)	0.458
SSTI	0.000 (0,000, 0.000)	1.000
Bacteremia	1.218 (0.761, 1.950)	0.411

Categorical data are presented as frequencies (percentages), and parametric continuous data are presented as medians (interquartile ranges).

*SOFA score* sequential organ failure assessment score, *LODS score* logistic organ dysfunction system score, *SAPS II score* simplified acute physiology II score, *PH* potential of hydrogen, *PO2* arterial partial pressure of oxygen, *PCO2* arterial blood carbon dioxide partial pressure, *BUN* blood urea nitrogen, Creatinine serum creatinine, *TBIL* total bilirubin, *ALT* alanine aminotransferase, *AST* aspartate aminotransferase, *ALP* alkaline phosphatase, *WBC* white blood cell, *RDW* red blood cell distribution width, *MCV* mean corpuscular volume, *MCH* mean corpuscular hemoglobin, *MCHC* mean corpuscular hemoglobin concentration, *INR* international normalized ratio, *APTT* partial thromboplastin time, *SSTI* skin and soft tissue infection.

### Verification of nomogram predictability

**Figure 4:**
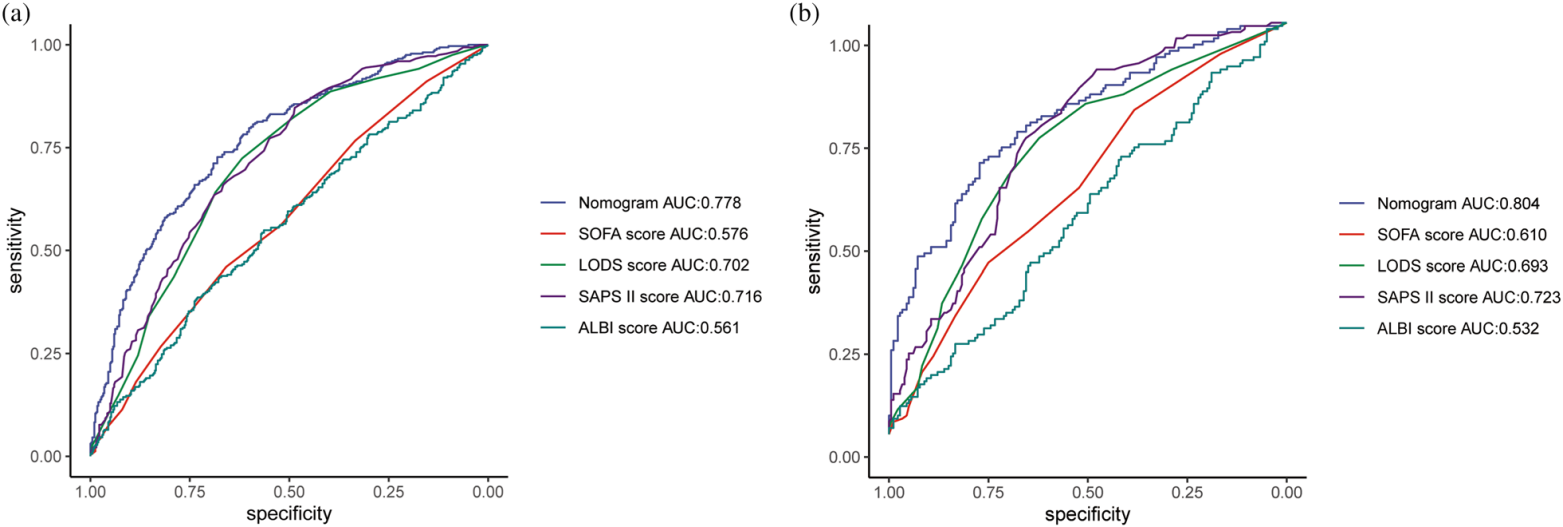
The ROC curve of the nomogram. (**a**) Training set; (**b**) validation set.

**Figure 5:**
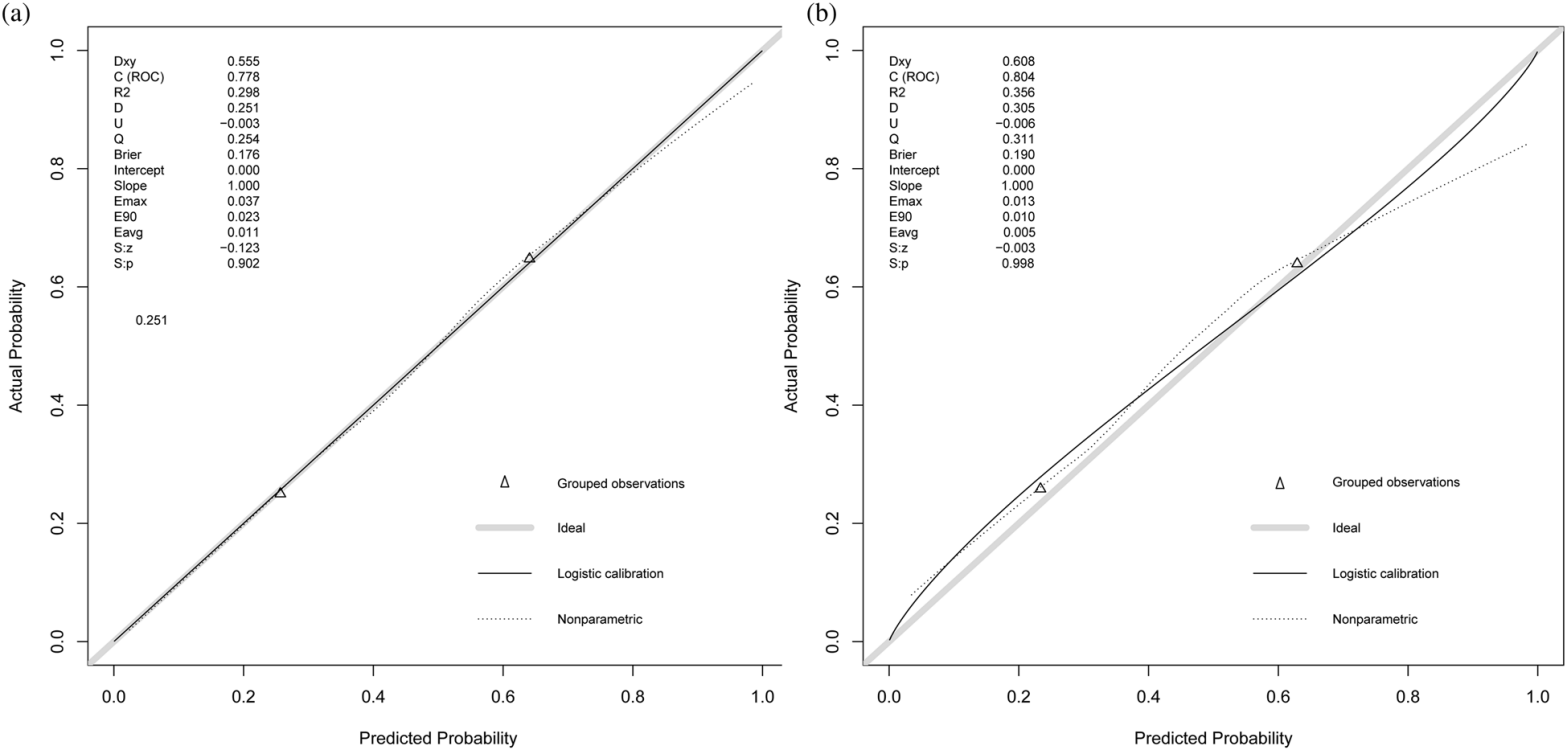
The calibration curve of the nomogram. (**a**) Training set; (**b**) validation set.

The nomogram was compared with SOFA, LODS, SAPS II and ALBI to monitor 90-day mortality in SALI patients. The AUROC for the training and validation groups of the nomogram was 0.778 (95% CI 0.730–0.799) and 0.804 (95% CI 0.713–0.820), respectively, which were higher than several other scores (Table 3, Fig. 4). In the training set, R2 score = 0.298, Brier score = 0.176, and in the validation set, R2 score = 0.356, Brier score = 0.190. There was also no linear correlation between the continuous variables in the model (Supplementary Fig. 2). The nomogram generally showed excellent consistency with the actual 90-day death rates depending on the calibration curves in the predicted and realistic death rates (Fig. 5). In addition, the IDI and NRI index for the nomogram were also superior to the SOFA, LODS, SAPS II and ALBI scores in the training and validation groups, suggesting that the nomogram had a superior predictive probability for 90-day mortality prediction (Table 3).

**Table 3:** Comparison of models for predicting the 90-day mortality of SALI.

Predictive Mode	AUROC	P value	IDI	P value	NRI P value
Training set					
Nomogram	0.778 (0.730, 0.799)				
SOFA	0.576 (0.535, 0.618)	< 0.001	0.191 (0.162, 0.220)	< 0.001	0.370 (0.280, 0.460) < 0.001
LODS	0.702 (0.664, 0.740)	< 0.001	0.094 (0.046, 0.142)	< 0.001	0.205 (0.057, 0.352) < 0.001
SAPS II	0.716 (0.679, 0.753)	< 0.001	0.082 (0.049, 0.114)	< 0.001	0.191 (0.098, 0.285) < 0.001
ALBI	0.561 (0.519, 0.603)	0.004	0.204 (0.174, 0.234)	< 0.001	0.379 (0.312, 0.446) < 0.001
Validation set					
Nomogram	0.804 (0.713, 0.820)				
SOFA	0.610 (0.547, 0.673)	0.001	0.194 (0.147, 0.241)	< 0.001	0.388 (0.241, 0.536) < 0.001
LODS	0.693 (0.634, 0.753)	< 0.001	0.120 (0.070, 0.169)	< 0.001	0.205 (0.040, 0.326) < 0.001
SAPS II	0.723 (0.667, 0.779)	< 0.001	0.094 (0.046, 0.142)	< 0.001	0.183 (0.057, 0.352) < 0.001
ALBI	0.532 (0.467, 0.597)	0.003	0.217 (0.170, 0.265)	< 0.001	0.393 (0.290, 0.497) < 0.001

### Clinical use of the nomogram

**Figure 6:**
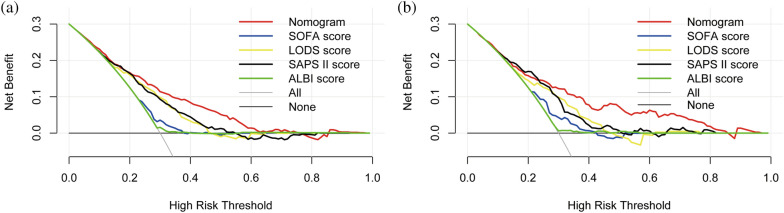
The decision curve analysis of the nomogram. (**a**) Training set; (**b**) validation set.

In the training set, nomogram-directed medical interventions could add more net benefit than the SOFA, LODS, SAPS II and ALBI scores when the threshold probability (PT) was 0.2–0.6 (Fig. 6a). In the validation set, obtaining more net benefit from nomogram-directed treatment than others was possible when the PT was between 0.3 and 0.8 (Fig. 6b). Simultaneously, the clinical benefit curves are given (Supplementary Fig. 3). The red line (number of high-risk people) shows the number of patients at high risk at each of the risk thresholds for 1000 patients, and the blue line (number of high-risk people with outcomes) shows truthful positive patient counts below the risk threshold.

### Ninety-day mortality risk from the nomogram

The nomogram is a predictive model with excellent sensitivity, specificity and predictive value in identifying 90-day mortality in SALI patients (Supplementary Table 3).

## Discussion

Sepsis in the health care field has remained a problematic subject in general health services, as its mortality rate is high, ranging from 20 to 50%^22–24^. Hepatic failure is a component of MODS in sepsis and is often associated with a poor prognosis, although its exact incidence is unknown^25,26^. Liver injury can be associated with an exacerbation of multiple organ failure^27^.

The results of our research reported a morbidity rate of 7.91% (1039 of 12129 patients) and mortality rate of 44.10% (458 of 1039 patients) in patients with SALI, suggesting a lower incidence and higher death rate than the Liu et al.^15^ and Kobashi et al.^27^ clinical reports. We found that Liu’s team used the older data version 1.0 of MIMIC-IV, which only included liver function indicators in the SALI diagnostic criteria and did not include the more novel combined liver function and coagulation criteria, while that allowed the inclusion of SALI diagnosed any time within 48 h of hospitalization, but our study only included the first 24 h of ICU MIMIC-IV in version 2.0. Therefore, we hypothesized that these differences resulted in a lower patient rate but a higher mortality rate than the study by Liu’s team. Again, in the 2013 investigation by Professor Kobashi, a wider scale of sepsis 2.0 criteria was used, and the standards for inclusion in the SALI group only covered liver function, without coagulation. The standards failed to assess more comprehensively the outcome of the inherent injury and the subsequent injury caused by SALI and therefore also reached the same conclusion as Liu’s team. However, these teams are all aware that a tool is not enough to predict prognosis in sepsis patients and liver injury patients, consistent with our idea.

In this article, the 10 indicators are age, the use of vasopressors, mean arterial pressure, mean SpO2, lactate maximum, BUN maximum, TBIL maximum, albumin minimum, RDW maximum and APTT maximum, from which we found that the BUN maximum has the largest weight. In accordance with its contribution to the nomogram, BUN level was the maximum factor of 90-day mortality in SALI patients. BUN levels are strongly correlated with mortality in patients with sepsis, and sepsis patients have a higher rate of death when their BUN levels increase^28–31^. A large study^31^ found that this association disappeared at the 41.1 mg/dL turning point. For every 10 mg/dL increase in BUN level, sepsis patients had a 29.8% increase in 30-day mortality, while patients with a BUN level ≥ 41.1 mg/dL experienced only a 4.5% increase in 30-day mortality. The BUN level can be utilized as an easy-to-use and rapid measure for early identification in sepsis patients because early and effective management is crucial in this condition. In a study of 2917 patients with sepsis, BUN level (1.08 [1.07–1.09]) was a strong factor of the incidence of AKI with sepsis, and the most sensitive indicator of AKI occurrence was BUN level^32^. Although the mechanism by which elevated BUN levels can contribute to the poor prognosis associated with sepsis is not clearly defined, there are still several possible reasons that can be explained. Patients in the severe stage are in a hyperproteolytic metabolic state^33^, and BUN levels increase when protein is excessively catabolic or the renal filtration rate is reduced. Therefore, BUN levels can play an important role in body protein catabolism^34^ and are a sign of renal damage. The rate of protein catabolism is significantly increased in sepsis patients^35^, and sepsis is usually accompanied by acute kidney injury^36^. These factors can contribute to elevated BUN levels in patients with sepsis. The BUN level is more widely used in sepsis and other areas of sepsis and is being used for the first time in patients with SALI and was first reported in adult SALI patients in our study.

There are several other variables in the nomogram that play an important role. A cohort study showed that age was an independent factor in the incidence of SALI patients. Among subjects not over 39 years old, the proportions of “cholestatic”, “hepatocellular”, and “shock liver” disease were 22.2%, 66.6%, and 11.1%, respectively. Additionally, among subjects who were at or over 40 years of age, these percentages were 51.5%, 15.9%, and 32.6%, respectively^27^. In a recent study, a scholar referred to age as an important component of mortality involved in SALI patients^15^. With the increasing severity of sepsis disease, a large proportion of patients have circulatory instability and require ascending agents to maintain hemodynamic stability^37–39^, yet the inclusion of vasopressors and mean arterial pressure were first reported in sepsis-associated liver injury in our study. Blood lactate, an indicator of tissue perfusion, is often used to provide feedback on survival in patients with sepsis^40,41^, and some high-quality studies on the effect of lactate on mortality in SALI patients have been identified only in children^42,43^; this is the first report in adult SALI patients. Some of the other variables, such as TBIL, albumin, RDW and APTT, which are frequently used in research to predict sepsis and sepsis-associated injury^14,17,18,44,45^, are rarely mentioned in studies of sepsis-associated liver injury, especially in adult reports^15^. Our findings suggest that all of these variables independently predict mortality from sepsis-associated liver injury.

Initially, clinical prediction models were used for oncology patients^46–48^, and as an increasing number of researchers continue to develop their understanding of clinical prediction models, they are commonly used in critical illness^49,50^. There is no authoritative standard for the prognostic assessment of patients with sepsis-associated liver injury to date, and there are few large data studies with a very strong evidence-based medical basis. Our study contains a visual nomogram based on 10 clinically readily available and commonly used parameters that were extracted from a large database called MIMIC-IV, and the efficiency of the current nomogram underwent thorough comprehensive evaluation and internal validation.

Professor Fragaki stated that the ALBI score is more appropriate for the identification and prognostic evaluation of early-onset liver dysfunction in a recent study^51^; therefore, we chose the ALBI score to assess liver function in this article. The SOFA score, LODS score and SAPS II score are widely used in mortality risk analysis and prognosis assessment of patients with sepsis^17,45,52,53^. It is rare to use a model to predict both sepsis and liver injury at the same time, except in the few reports where the level of evidence is not high^15^. Nevertheless, the validity of these scores in prescribing the risk of 90-day mortality in SALI patients still remains unknown. To compare these scores, we evaluated the hypothesized nomogram’s predictive accuracy with various widely used clinical scores, including the SOFA, LODS, SAPS II, and ALBI scores, based on the AUROC. The nomogram performed best in all of the tools. The result that the nomogram could successfully distinguish between true positive patients at high risk of 90-day mortality in both the training and validation sets was further evidenced by DCA curves, IDI, and NRI indices. The nomogram in this case performed better in differentiating the risk of 90-day mortality, as supported by the high C-index (0.778) in the training set and the C-index (0.804) in the validation set, as well as by acceptable calibration. By developing the scale score, the change in each variable is described from the forest plot, and then the total score is computed to predict the possibility that an event will occur.

Several limitations include the following: (1) There is a lack of a definitive definition of SALI. (2) The confounding factors can occur with the inclusion of each variable, which can affect the results because this study is retrospective. (3) According to the definition of sepsis, the addition of specific pathogenic culture results might have improved the predictive strength of the model. Our study only contains parameters related to the first day in the ICU, and it might have been better to have dynamic, continuous observational analysis data on indicators during the ICU stay. (4) When we imported data from the MIMIC-IV database, we found that many variables were missing, and even some missing data were greater than 50%, and these parameters might have an impact on our findings. (5) The MIMIC-IV database is only a single-center study. In the future, multicenter research can be performed in different countries and regions with different economic levels, and external databases can be used for validation. These are endeavors we will pursue in the future.

## Conclusion

The nomogram is a comprehensive performance of 10 indicators that have been tested for meaningfulness, including age, usage of vasopressor on the first day of ICU stay, mean arterial pressure, mean spo2, lactate maximum, BUN maximum, TBIL maximum, albumin minimum, RDW maximum and APTT maximum, which can be easily used for accurate measurement of 90-day mortality in SALI patients. This nomogram may be extremely valuable in controlling the progression of SALI when usual measures have been conducted and in ultimately improving the prognosis of SALI patients.

## Supplementary Information

Supplementary Information.

Supplementary Figure 1.

Supplementary Figure 2.

Supplementary Figure 3.

Supplementary Table 1.

Supplementary Table 2.

Supplementary Table 3.

## Data Availability

The Medical Information Mart for Intensive Care IV (MIMIC-IV) database is a public database that incorporates detailed patient information from patients hospitalized at Beth Israel Deaconess Hospital (Bowers, Massachusetts, USA) between 2008 and 2019. A retrospective study was performed using the MIMIC-IV (v 2.21) database. The database is constantly updated with the latest version (v2.21) released on 12 July 2022, and more data have been added to increase the comprehensiveness of the data. The datasets generated during the current study are available in the MIMIC repository, [https://mimic.mit.edu/]. The data that support the findings of this study are available from MIMIC but restrictions apply to the availability of these data, which were used under license for the current study, and so are not publicly available. Data are however available from the authors upon reasonable request and with permission of the National Institutes of Health (NIH).
