# Supplementary material for: Development of a nomogram for predicting 90-day mortality in patients with sepsis-associated liver injury
Source: Sci Rep. 2023 Mar 4;13:3662. doi: 10.1038/s41598-023-30235-5 (PMC9985651; doi:10.1038/s41598-023-30235-5)
Supplement: Supplementary file 2 — Supplementary Figure 1. [file 41598_2023_30235_MOESM2_ESM.docx]

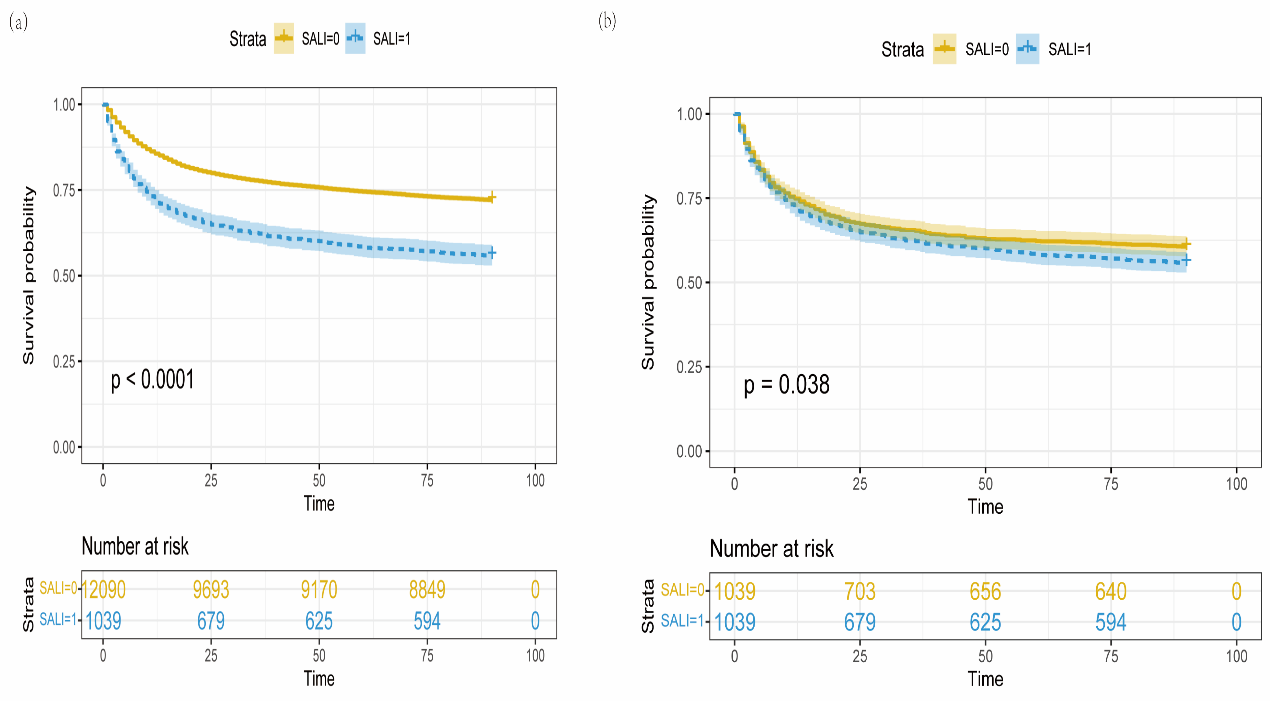


Supplementary Figure 1. The Kaplan-Meier’s survival estimated of the 90-day survival probability of SALI and non-SALI patients. Results showed that the 90-day survival of SALI patients were significantly lower than that of non- SALI patients (Log-rank p <0.001).(a)

The Kaplan-Meier’s survival estimated of the 90-day survival probability of SALI and non-SALI patients after PSM. Results showed that the 90-day survival of SALI patients were significantly lower than that of non- SALI patients (Log-rank p=0.038).(b)
