# Supplementary material for: Development of a nomogram for predicting 90-day mortality in patients with sepsis-associated liver injury
Source: Sci Rep. 2023 Mar 4;13:3662. doi: 10.1038/s41598-023-30235-5 (PMC9985651; doi:10.1038/s41598-023-30235-5)
Supplement: Supplementary file 3 — Supplementary Figure 2. [file 41598_2023_30235_MOESM3_ESM.docx]

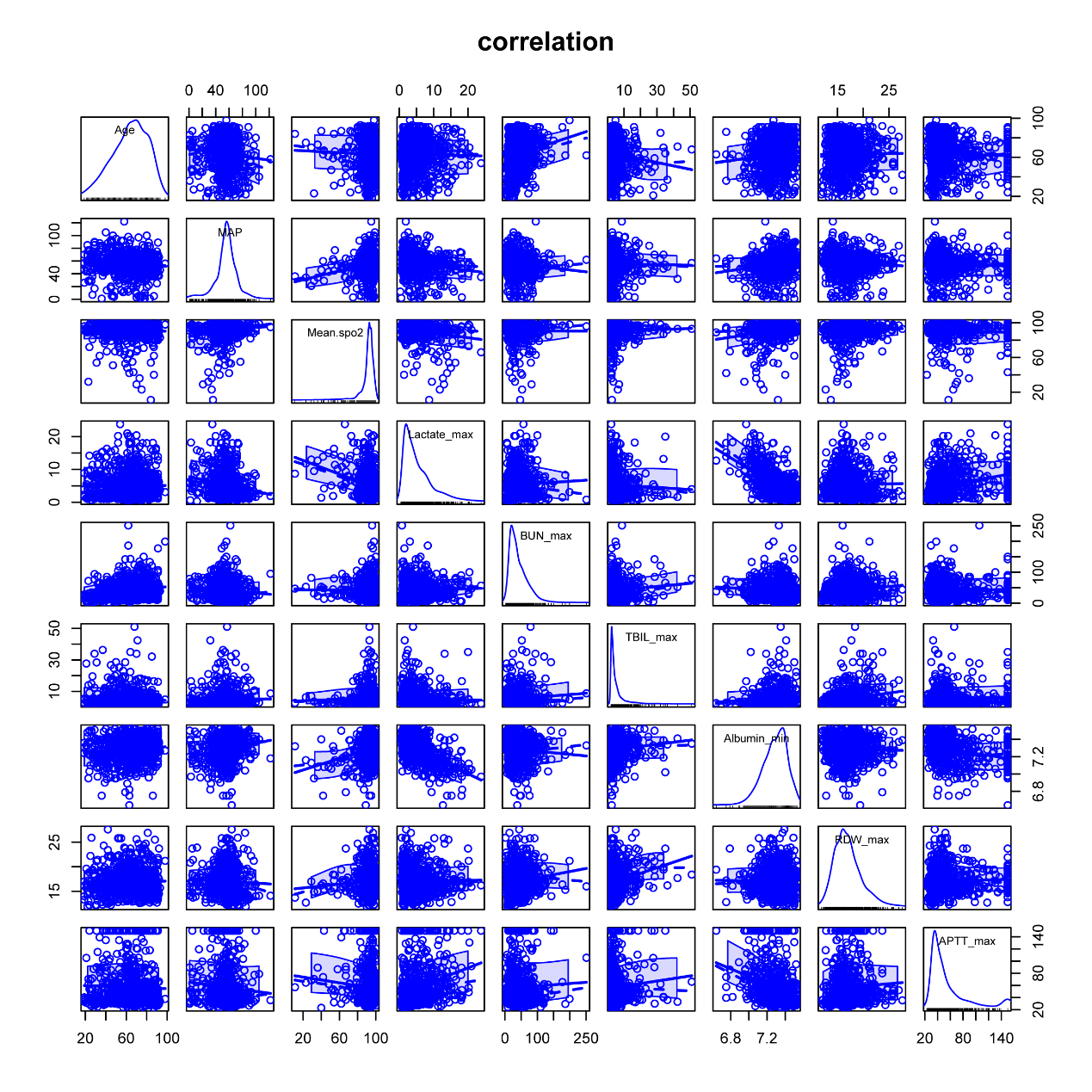


Supplementary Figure 2. The figure exhibited that no linear correlation was existed among the continuous variables, indicating that collinearity was not existed in the multivariable logistic regression.
