# Supplementary material for: Development of a nomogram for predicting 90-day mortality in patients with sepsis-associated liver injury
Source: Sci Rep. 2023 Mar 4;13:3662. doi: 10.1038/s41598-023-30235-5 (PMC9985651; doi:10.1038/s41598-023-30235-5)
Supplement: Supplementary file 4 — Supplementary Figure 3. [file 41598_2023_30235_MOESM4_ESM.docx]

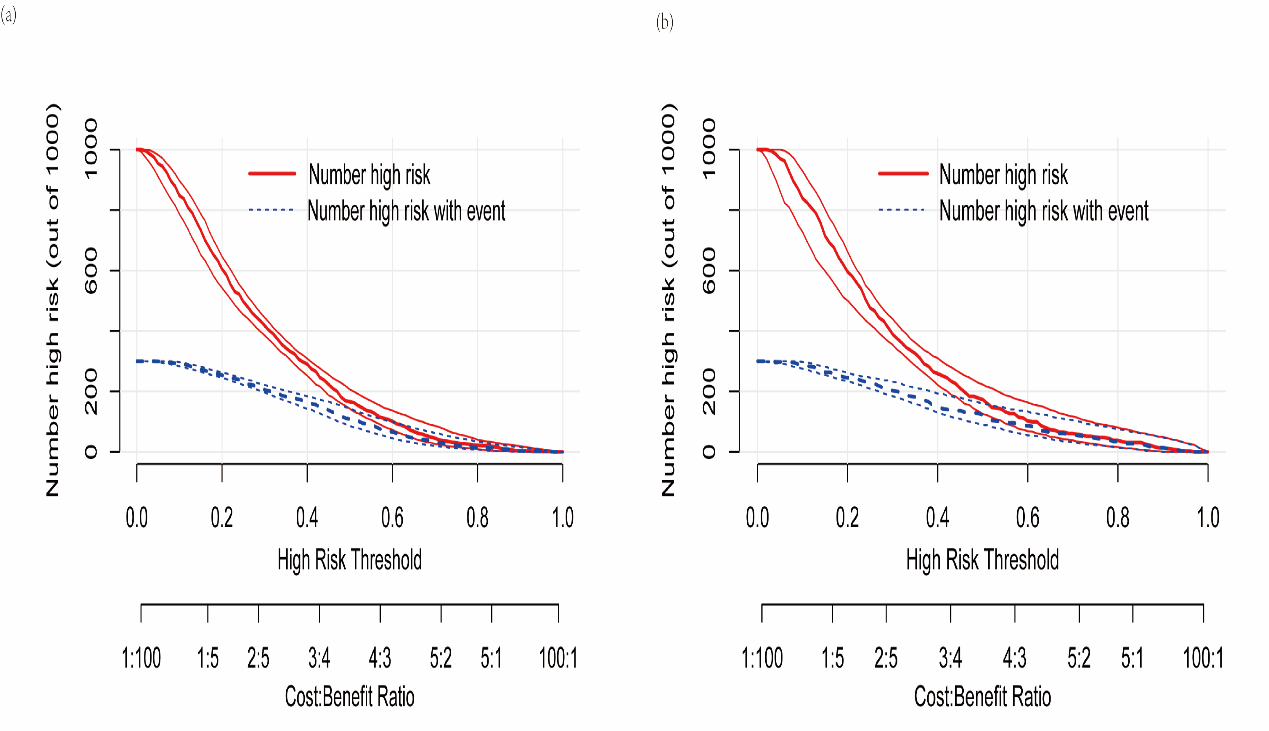


Supplementary Figure 3. The clinical impact curve of the nomogram, in which red solid curve indicates the number of people who are classified as high risk by the nomogram at each threshold probability; the blue dashed curve showed the number of true positive patients under each risk threshold. (a) Training set; (b) Validation set
