# Supplementary material for: Development of a nomogram for predicting 90-day mortality in patients with sepsis-associated liver injury
Source: Sci Rep. 2023 Mar 4;13:3662. doi: 10.1038/s41598-023-30235-5 (PMC9985651; doi:10.1038/s41598-023-30235-5)
Supplement: Supplementary file 5 — Supplementary Table 1. [file 41598_2023_30235_MOESM5_ESM.docx]

Supplementary Table 1 Factors independently associated with 90-day mortality of patients with sepsis by multivariate logistic regression analysis.

| **Variable** | **OR(95% CI)** | **P value** |  |
| --- | --- | --- | --- |
| SALI | 1.158(0.970,1.240) | 0.034 |  |
| Age, years | 1.036(1.031,1.041) | <0.001 |  |
| Myocardial infarct | 1.194(1.012,1.409) | 0.036 |  |
| Cerebrovascular disease | 1.936(1.604,2.337) | <0.001 |  |
| Diabetes | 0.747(0.597,0.934) | 0.011 |  |
| Paraplegia | 1.604(1.200,2.142) | 0.001 |  |
| First day CRRT, n (%)^a^ | 1.578(1.174,2.122) | 0.003 |  |
| First day MV, n (%)^a^ | 1.034(0.989,1.192) | <0.001 |  |
| Mean heart rate (min−1) | 1.006(1.003,1.009) | 0.001 |  |
| Mean arterial pressure (mmHg) | 0.995(0.990,1.000) | 0.033 |  |
| Mean temperature (°C) | 0.798(0.742,0.858) | <0.001 |  |
| Lactate_max, (mmol/L) | 1.161(1.098,1.229) | <0.001 |  |
| BUN_max, (mg/dl) | 1.008(1.004,1.011) | <0.001 |  |
| Creatinine _max, (mg/dl) | 0.913(0.861,0.962) | 0.002 |  |
| ALP_max(U/L) | 1.001(1.000,1.002) | <0.001 |  |
| WBC_max, (K/uL) | 1.001(1.003,1.108) | 0.005 |  |
| RDW_max, (%) | 1.165(1.123,1.208) | <0.001 |  |
| INR_max | 1.056(1.006,1.109) | 0.027 |  |
| APTT_max, (s) | 1.003(1.001,1.005) | 0.001 |  |

SALI, sepsis-associated liver injury; ^a^First day of ICU usage means the number of ICU uses on the first day; CRRT, continuous renal replacement therapy; MV, mechanical ventilation; BUN, blood urea nitrogen; Creatinine, serum creatinine; ALP, alkaline phosphatase; WBC, white blood cell; RDW, red blood cell distribution width; INR, international normalized ratio; APTT, partial thromboplastin time.
