# Supplementary material for: Development of a nomogram for predicting 90-day mortality in patients with sepsis-associated liver injury
Source: Sci Rep. 2023 Mar 4;13:3662. doi: 10.1038/s41598-023-30235-5 (PMC9985651; doi:10.1038/s41598-023-30235-5)
Supplement: Supplementary file 6 — Supplementary Table 2. [file 41598_2023_30235_MOESM6_ESM.docx]

Supplementary Table 2 The characteristics of SALI patients in the training set and validation set.

| **Variable** | **SALI patients**  **(n = 1039)** | **Training set (n=727)** | **Validation set (n=312)** | **P value** |
| --- | --- | --- | --- | --- |
| Age, years | 65(51,77) | 65(52,77) | 65(50.25,77) | 0.790 |
| Gender, male | 628 (60.44) | 450 (61.90) | 178 (57.05) | 0.163 |
| **Comorbidity, n (%)** |  |  |  |  |
| Myocardial infarct | 181(17.42) | 130(17.88) | 51(16.35) | 0.611 |
| Congestive heart failure | 442(42.54) | 302(41.54) | 140(44.87) | 0.354 |
| Chronic pulmonary disease | 251(24.16) | 183(25.17) | 68(21.79) | 0.277 |
| Peripheral vascular disease | 150(14.44) | 100(13.76) | 50(16.03) | 0.391 |
| Cerebrovascular disease | 86 (8.28) | 66(9.08) | 20(6.41) | 0.191 |
| Diabetes | 92(8.85) | 72(9.90) | 20(6.41) | 0.090 |
| Rheumatic disease | 36(3.46) | 25(3.44) | 11(3.53) | 1.000 |
| Peptic ulcer | 41(3.95) | 27(3.71) | 14(4.49) | 0.680 |
| Paraplegia | 29(2.79) | 15(2.06) | 14(4.49) | 0.049 |
| Renal disease | 276(26.56) | 193(26.55) | 83(26.60) | 1.000 |
| **Severity score** |  |  |  |  |
| SOFA score | 5.00(3.00,7.00) | 5.00(3.00,7.00) | 5.00(3.00,7.00) | 0.904 |
| LODS score | 8.00(5.00,11.00) | 8.00(5.00,11.00) | 8.00(5.00,11.00) | 0.343 |
| SAPS II score | 46.00(35.00,58.00) | 46.00(35.00,57.00) | 45.00(34.00,59) | 0.508 |
| **First day of ICU usage^a^** |  |  |  |  |
| First day CRRT, n (%) | 97(9.34) | 64(8.80) | 33(10.58) | 0.433 |
| First day vasopressor, n (%) | 677(65.16) | 482(66.30) | 195(62.50) | 0.268 |
| First day MV, n (%) | 512(49.28) | 361(49.66) | 151(48.40) | 0.804 |
| **Vital signs^b^** |  |  |  |  |
| Mean heart rate (min−1) | 108.00(93.00,124.00) | 108.00(93.00,124.00) | 109.00(92.00,125.75) | 0.981 |
| Mean arterial pressure (mmHg) | 57.00(49.00,65.00) | 56.00(49.00,65.00) | 58.00(50.00,66.00) | 0.063 |
| Mean respiratory rate (min−1) | 28.00(24.00,33.00) | 28.00(24.00,33.00) | 28.00(24.00,33.00) | 0.919 |
| Mean temperature (°C) | 37.06(36.72,37.67) | 37.11(36.74,37.67) | 37.00(36.67,37.61) | 0.098 |
| Mean spo2 (%) | 93.00(90.00,95.00) | 93.00(90.00,95.00) | 93.00(89.00,95.75) | 0.558 |
| **Blood gas analysis^c^** |  |  |  |  |
| PH-min | 7.30(7.19,7.38) | 7.30(7.19,7.39) | 7.29(7.20,7.36) | 0.121 |
| PO2-min, (mmHg) | 84.00(67.00,106.00) | 84.00(68.00,106.00) | 84.00(64.00,107.00) | 0.957 |
| PCO2-max, (mmHg) | 41.00(35.00,50.00) | 41.00(30.00,50.00) | 42.00(35.25,50.00) | 0.271 |
| Lactate_max, (mmol/L) | 4.00(2.10,7.30) | 4.00(2.10,7.20) | 4.00(2.12,7.30) | 0.903 |
| Bicarbonate_min, (mEq/L) | 18.00(15.00,22.00) | 18.00(15.00,22.00) | 18.00(15.00,22.00) | 0.791 |
| **Laboratory tests^c^** |  |  |  |  |
| Glucose_max, (mg/dl) | 152.00(118.00,216.00) | 153.00(118.00,217.00) | 152.00(121.00,212.00) | 0.875 |
| Aniongap_max | 19.00(16.00,24.00) | 19.00(16.00,24.00) | 19.00(16.00,24.00) | 0.700 |
| Chloride_max, (mEq/L) | 105.00(100.00,110.00) | 105.00(100.00,110.00) | 104.00(100.00,109.00) | 0.261 |
| Potassium_max, (K/uL) | 4.60(4.10,5.20) | 4.60(4.20,5.20) | 4.60(4.10,5.20) | 0.800 |
| Sodium_min, (mEq/L) | 136.00(132.00,139.00) | 135.00(132.00,139.00) | 136.00(132.00,138.00) | 0.806 |
| Calcium_min, (mg/dl) | 7.70(7.10,8.20) | 7.70(7.20,8.20) | 7.75(7.20,8.28) | 0.586 |
| **Indicators for kidney function^c^** |  |  |  |  |
| BUN_max, (mg/dl) | 28.00(18.00,47.00) | 34.00(20.00,55.00) | 32.00(19.00,55.00) | 0.415 |
| Creatinine _max, (mg/dl) | 1.40(0.90,2.30) | 1.70(1.10,2.70) | 1.70(1.10,2.67) | 0.536 |
| **Indicators for liver function^c^** |  |  |  |  |
| TBIL_max ((mg/dl) | 3.60(2.60,5.60) | 3.60(2.60,5.60) | 3.80(2.70,6.00) | 0.384 |
| ALT_max (U/L) | 106.00(36.00,434.00) | 98.00(36.00,420.00) | 117.00(36.25,521.75) | 0.265 |
| AST_max (U/L) | 163.00(69.00,643.00) | 160.00(67.00,614.00) | 176.50(72.50,689.75) | 0.339 |
| ALP_max(U/L) | 132.00(80.00,229.00) | 134.00(81.00,233.00) | 124.00(79.25,218.25) | 0.443 |
| Albumin_min (g/dl) | 2.80(2.30,3.20) | 2.80(2.20,3.30) | 2.80(2.40,3.20) | 0.674 |
| **Blood routine examination^c^** |  |  |  |  |
| WBC_max, (K/uL) | 15.80(10.40,22.40) | 15.80(10.50,22.50) | 15.80(10.15,22.30) | 0.612 |
| Hemoglobin_min (g/dl) | 9.10(7.70,10.90) | 9.00(7.70,10.90) | 9.30(7.70,11.08) | 0.344 |
| Platelet _min (K/uL) | 111.00(66.00,168.00) | 112.00(66.00, 168.00) | 109.00(62.00,171.00) | 0.597 |
| RDW_max, (%) | 16.60(14.90,18.40) | 16.70(15.10,18.50) | 16.60(14.83,18.40) | 0.292 |
| MCV_min, (fL) | 91.00(86.00,96.00) | 91.00(86.00,96.00) | 91.00(86.00,96.00) | 0.856 |
| MCH_min, (pg) | 30.30(28.70,31.80) | 30.20(28.70,31.80) | 30.50(28.63,31.98) | 0.193 |
| MCHC_min, (g/L) | 33.10(31.90,34.10) | 33.10(31.90,34.10) | 33.00(32.00,34.20) | 0.708 |
| **Indicators for coagulation function^c^** |  |  |  |  |
| INR_max | 2.20(1.80,3.20) | 2.20(1.80,3.10) | 2.20(1.80,3.38) | 0.317 |
| APTT_max, (s) | 44.60(35.70,65.10) | 44.70(35.20,64.20) | 44.20(37.12,72.12) | 0.087 |
| **Infection site, n (%)** |  |  |  |  |
| Lung | 94(9.05) | 62(8.53) | 32(10.26) | 0.440 |
| Abdominal | 12(1.15) | 9(1.24) | 3(0.96) | 0.485 |
| Urinary tract | 7(0.67) | 6(0.83) | 1(0.32) | 0.618 |
| SSTI | 1(0.01) | 1(0.14) | 0(0.00) | <0.001 |
| Bacteremia | 925(89.03) | 642(88.31) | 283(90.71) | 0.954 |
| **Outcome** |  |  |  |  |
| ICU stay time, days | 4.17(2.21,8.88) | 4.25(2.33,9.08) | 3.81(2.05,7.70) | 0.118 |
| Hospital stay time, days | 11.00(6.00,20.00) | 11.00(6.00,21.00) | 10.00(6.00,20.00) | 0.306 |
| 90- day mortality, n (%) | 581(55.92) | 401(55.16) | 180(57.69) | 0.493 |
| MV, n (%) | 611(58.81) | 437(60.11) | 174(55.77) | 0.217 |
| CRRT, n (%) | 218(20.98) | 155(21.32) | 63(20.19) | 0.744 |
| Vasopressor, n (%) | 730(70.26) | 520(71.53) | 210(67.31) | 0.197 |

Categorical data are presented as frequencies (percentages), and parametric continuous data are presented as medians (interquartile ranges).

SOFA score, Sequential Organ Failure Assessment score; LODS score, Logistic Organ Dysfunction System score; SAPS II score, simplified acute physiology II score.

^a^First day of ICU usage means the number of ICU uses on the first day; CRRT, continuous renal replacement therapy; MV, mechanical ventilation.

^b^Vital signs were calculated as the mean value during each patient's first 24 hours after ICU admission.

^c^The laboratory tests recorded the worst value during each patient's first 24 hours from ICU admission.

PH, potential of hydrogen; PO2, arterial partial pressure of oxygen; PCO2, arterial blood carbon dioxide partial pressure; BUN, blood urea nitrogen; Creatinine, serum creatinine; TBIL, total bilirubin; ALT, alanine aminotransferase; AST, aspartate aminotransferase; ALP, alkaline phosphatase; WBC, white blood cell; RDW, red blood cell distribution width; MCV, mean corpuscular volume; MCH, mean corpuscular hemoglobin; MCHC, mean corpuscular hemoglobin concentration; INR, international normalized ratio; APTT, partial thromboplastin time; SSTI, skin and soft tissue infection.
