# Supplementary material for: Development of a nomogram for predicting 90-day mortality in patients with sepsis-associated liver injury
Source: Sci Rep. 2023 Mar 4;13:3662. doi: 10.1038/s41598-023-30235-5 (PMC9985651; doi:10.1038/s41598-023-30235-5)
Supplement: Supplementary file 7 — Supplementary Table 3. [file 41598_2023_30235_MOESM7_ESM.doc]

Supplementary Table 3 Accuracy of the Nomogram for predicting the risk of 90-day mortality in SALI patients.

| **Variable** | **Value(95% CI)** | |
| --- | --- | --- |
|  | **Training set** | **Testing set** |
| AUROC | 0.778(0.730, 0.799) | 0.804(0.713, 0.820) |
| Sensitivity, % | 0.730 (0.561, 0.841) | 0.694 (0.484, 0.919) |
| Specificity, % | 0.693 (0.571, 0.845) | 0.805 (0.529, 0.93) |
| Positive predictive value, % | 0.660 (0.605, 0.757) | 0.698 (0.560, 0.861) |
| Negative predictive value, % | 0.759 (0.698, 0.822) | 0.784 (0.706, 0.907) |

AUROC area under the receiver operating characteristic curve.
